# Food Production as a challenge for One Health Approach and Public Health

**DOI:** 10.1093/eurpub/ckac129.219

**Published:** 2022-10-25

**Authors:** M Jevtic, C Bouland

**Affiliations:** 1 Faculty of Medicine, University of Novi Sad, Serbia, Serbia; 2 Institute of Public Health of Vojvodina, Novi Sad, Serbia, Serbia; 3 Université Libre de Bruxelles, Research Center, School of Public Health, Brussels, Belgium

## Abstract

A sustainable food production is essential for human sustainability. According to available data, the world's diets and food habits must evolve and change intensely. Nowadays, more than 800 million people suffer from deficient food. On the other hand, more consumers have an unhealthy diet contributing to premature death, obesity, food related non-communicable diseases. Diets must provide an appropriate calorie intake, different of plant-based food, low amounts of animal-based food, unsaturated fats, vitamins and minerals (rather than saturated, refined grains, highly processed foods, and added sugars). It is needed adjustment to various agro-systems, cultural traditions, and individual dietary preferences. However, production must also be sustainably adjusted to meet the global population's growing food demands, as well as using the doughnut economy principle. A sustainable food production requires a One Health approach, and also evolving towards a decarbonized agricultural production by eliminating the use of fossil fuels and land use change losses of CO2 in agriculture. Policies to encourage people to choose healthy diets are needed. Those should include the improvement of the availability and accessibility to healthy food through improved logistics and storage, increased food security, policies that promote buying from sustainable sources, as well as reducing food waste. The mindset change regarding to the agro-system, food production and food usage in a healthy way at individual level, community level, national level and global level are essential for sustainable human population. Healthy eating should be the highest priority of food consumers, as well as its mindset shift from ‘live to eat’ to ‘eat to live’. Consumers should feel safe and healthy, but also think about future generations, respecting agriculture and food production. Sustainable, equitable, healthier, and more inclusive food systems have the power to catalyze the achievement of all 17 SDGs by 2030.

